# The methods and baseline characteristics of a VA randomized controlled study evaluating supported employment provided in primary care patient aligned care teams

**DOI:** 10.1186/s12874-020-0919-1

**Published:** 2020-02-17

**Authors:** Lori L. Davis, Catherine M. Blansett, Mercy N. Mumba, David MacVicar, Richard Toscano, Patricia Pilkinton, Whitney Gay, Al Bartolucci

**Affiliations:** 1 Tuscaloosa VA Medical Center, 3701 Loop Road East (151), Tuscaloosa, AL 35404 USA; 2 Department of Psychiatry, University of Alabama School of Medicine, Birmingham, AL USA; 3 Capstone College of Nursing, University of Alabama, Tuscaloosa, AL USA; 4 Department of Biostatistics, University of Alabama at Birmingham, Birmingham, AL USA

**Keywords:** Individual placement and support, Patient aligned care team, Supported employment, Transitional work, Vocational rehabilitation, Unemployment, Employment, Veterans, Primary care

## Abstract

**Background:**

This article describes the design and baseline sample of a single-site trial comparing Individual Placement and Support (IPS) supported employment delivered within a Veterans Health Administration (VHA) primary care Patient Aligned Care Team (PACT) to treatment-as-usual vocational rehabilitation (TAU-VR) that includes transitional work.

**Methods:**

Unemployed U.S. military veterans receiving care in a VHA PACT who were seeking competitive work, otherwise eligible for vocational rehabilitation, and diagnosed with a mental health condition other than a psychotic or bipolar I disorder were prospectively randomized to receive either IPS or TAU-VR. Employment outcomes and measures of quality of life, self-esteem, and community reintegration are being collected for 12 months.

**Results:**

The participant sample (*n* = 119) is comprised of 17.6% female, 73.1% African-Americans, and 1.7% Hispanic. Average age is 38.2 (*SD* ± 8.41) years; 80.7% served in the military since 2001; 78% are receiving or applying for U.S. Department of Veterans Affairs (VA) service-connected disability; 26.9% have not held a competitive job in the past 3 years; and the average length of pre-randomization unemployment is 1.4 (*SD* ± 2.3) years.

**Conclusions:**

Unique design features include evaluating the efficacy of evidenced-based IPS within the primary care setting, having broad diagnostic eligibility, and defining the primary outcome criterion as “steady employment”, i.e. holding a competitive job for ≥26 weeks of the 12-month follow-up period. The findings illustrate the characteristics of a primary care veteran sample in need of employment services.

**Trial registration:**

www.clinicaltrials.gov Identifier: NCT02400736.

## Background

Over the past three decades, controlled studies of the evidence-based model of supported employment, called Individual Placement and Support (IPS), have yielded remarkably robust and consistent employment outcomes [1], outpacing comparison groups by as much as 3 times the employment rate and having advantages that are durable over extended periods of time [2, 3]. These studies have focused IPS services on individuals with a serious mental illness (SMI; i.e. schizophrenia, schizoaffective, bipolar I disorder, and major depression with psychotic features). Recent studies have proven that IPS is effective for veterans with posttraumatic stress disorder (PTSD) [ 4–6].

In 2004, the Veterans Health Administration (VHA) disseminated IPS as a service for veterans with an SMI diagnosis [7, 8]. However, transitioning service members and veterans struggling with unemployment have a broad spectrum of mental disorders that include anxiety, depression, and substance use [9–11]. In VA settings, IPS supported employment services historically have been prioritized for veterans with an SMI diagnosis, thus, excluding most VA consumers. In fact, research shows that very few recently-returning veterans with PTSD, traumatic brain injury, depression or substance abuse access VA vocational rehabilitation or supported employment services [12–14]. Additionally, veterans who are returning from recent deployments often delay seeking treatment in a mental health setting due to the stigma and fear of not being able to obtain employment if psychiatric needs are disclosed, thus, potentially delaying engagement in much needed vocational rehabilitation.

IPS has been studied in mental health settings and has generally limited enrollment to a confined diagnostic category rather than a broad range of non-SMI diagnoses that include major depressive, anxiety, and substance use disorders as the participant’s primary problem. In an innovative approach, this study breaks from the traditional design features of limiting inclusion to a diagnostic category and of integrating services within the mental health treatment setting. This study includes veterans with a broad range of non-SMI mental health diagnoses and evaluates the efficacy of IPS when provided in a primary care setting, specifically a Patient Aligned Care Team (PACT). The PACT is an innovation of primary care teams stemming directly from a patient-centered medical home model [15]. A PACT provides accessible, coordinated, and patient-centered care, and is managed by primary care providers with the colocation and involvement of mental health specialists. A PACT allows a patient to have a more active role in his/her health care and to access integrated care from one clinic. The PACT model is associated with increased quality improvement, patient satisfaction, and decreased hospital costs [15].

The specific objective of this prospective, randomized, controlled study is to determine the efficacy of IPS when delivered within a VA primary care PACT. The hypotheses to be tested are that, compared to a treatment-as-usual vocational rehabilitation (TAU-VR; defined below), IPS delivered within a PACT will result in a higher proportion of steady workers and greater improvement in quality of life, self-esteem, and community reintegration. In addition, this study evaluates whether IPS delivery within a PACT is feasible as reflected in the IPS fidelity scores (defined below) and customer satisfaction scores. The article also describes the baseline demographics and clinical characteristics of the enrolled veterans. Results of the study could potentially transform VHA delivery of IPS. Positive results would suggest that IPS employment services could be offered in a less stigmatizing primary care setting and possibly avoid unnecessary delays in care that result from a pre-vocational referral to a mental health clinic.

## Methods

### Ethics approval and consent to participate

This study is approved by the Tuscaloosa VA Medical Center Institutional Review Board (i.e. local human research subjects protections program’s ethics committee) and is conducted in accordance with the Declaration of Helsinki. All participants in the study provide written informed consent prior to participating in any study procedures. The study maintains Institutional Review Board approvals on an annual basis. The study is monitored by an independent Data and Safety Monitoring Board and has a Certificate of Confidentiality approved by the National Institute of Mental Health.

### Overview of design, aim, and hypotheses

Supported by VA Rehabilitation Research and Development, investigators at the Tuscaloosa VA Medical Center are currently conducting a single-site, randomized, controlled study to determine the effectiveness and feasibility of IPS when delivered within a primary care PACT in veterans who are unemployed and have a mental health diagnosis other than an SMI. The hypotheses are that, compared to TAU-VR (control), IPS delivered within a PACT will result in a higher rate of steady workers, defined as working ≥26 weeks in the 12-month follow-up period in a competitive job (primary); improved quality of life and self-esteem (secondary); very good veteran satisfaction and acceptability (tertiary); improved community re-integration (exploratory); and lower rates of high intensity crisis services (exploratory). Participants are recruited from outpatient primary care PACT clinics and community settings.

### Eligibility criteria

Veterans who are ≥19 years of age, receiving primary care in a PACT clinic, currently unemployed and interested in competitive employment, willing to be randomized to either existing TUA-VR or IPS services, and otherwise eligible for vocational rehabilitation services are eligible for study participation. Veterans must also be diagnosed with a current disabling or potentially disabling mental disorder, excluding a current diagnosis of schizophrenia, schizoaffective, bipolar I disorder, or major depression with psychotic features. Veterans who are unable to provide consent, unlikely to complete the study, actively suicidal or homicidal, have a diagnosis of dementia, or involved in another vocational study are ineligible for study participation. Veterans are not excluded based on gender, sexual orientation, race, ethnicity, or social class.

### Randomization and follow-up schedule

After signing informed consent, eligible participants are randomized to either IPS delivered in the PACT or TAU-VR services, which may include transitional work assignment at the Tuscaloosa VA Medical Center. Randomization utilizes a permuted block design of randomly varying block sizes. After randomization, the participants, providers, and outcomes assessors are openly aware of the treatment assignment. During the 12-month follow-up period, the participants are assessed monthly for employment outcomes and every two months for all other outcome assessments, except for the community reintegration assessment, which is conducted every four months.

### Clinical assessments

Information collected at baseline include demographics, military service history, employment history, status and type of housing, number of dependents, type of transportation, disability status, and family care burden. Baseline assessments include the MINI International Neuropsychiatric Interview Version 7 (MINI) to assess concurrent mental disorders for DSM-5 [16], the Cumulative Illness Rating Scale (CIRS) [17, 18] to asses general medical conditions and determine medical burden, and the Ohio State University Traumatic Brain Injury Identification Method – Short Form to assess lifetime history and severity of traumatic brain injury [19]. Repeated measures include: Rosenberg Self-Esteem Scale (RSES) [20], Quality of Life Inventory (QOLI) [21], Community Reintegration of Service Members (CRIS) [22], Symptom Checklist-90-Revised (SCL-90-R) [23], Client Satisfaction Questionnaire-8 (CSQ-8) [24], and Sheehan Suicidality Tracking Scale [25]. Additionally, the Clinical Research Coordinator (CRC) completes an Inventory of Crisis Events that assesses the number of emergency room visits, contacts with the legal system, and days of inpatient treatment, nights spent homeless, and days of substance misuse at baseline and follow-up visits.

### Employment outcome assessments

The primary outcome of “steady worker” is defined as holding competitive employment for at least 50% of the weeks during the 12-month follow-up (i.e., ≥ 26 of the 52 weeks), and “competitive employment” is defined as a job for salary, wages, or commission in a setting that is not set aside or enclaved (i.e., the same job could be held by people without mental illness or disability). Cash-based or transient jobs, such as yard work, babysitting, day labor, and military drill do not count as competitive employment. A week is recorded as “employed” if the participant holds a competitive job for ≥1 h/day for ≥1 day/week. Weeks do not have to be consecutively worked to count toward the threshold of “steady worker.” At baseline and reinforced at all follow-up visits, the participants are instructed to maintain a study-formatted Employment Calendar Diary, retain a copy of any pay records or tax forms, and bring the documents to the follow-up visits. In addition to reviewing these documents, the CRC reviews notes in the participant’s electronic medical record and conducts a semi-structured interview focused on employment activities. Based on these combined sources, the CRC records the following employment data for each week: 1) did the participant work for pay (Yes, No, Unknown); 2) type of work (transitional work, competitive, or other); 3) type of job(s) coded using the Hollingshead Categories from the Addictions Severity Index [26]; 4) number of days worked, 5) number of hours worked, 6) gross income earned, and 7) whether the job was new for each referenced week.

### Patient aligned care team (PACT) setting

PACT is a patient-driven, team-based approach to deliver efficient, comprehensive and continuous care that includes these domains: *Patient-driven:* Medical care is focused on the person rather than the condition or disease. The patients’ needs and preferences are the centerpiece of a partnership among the primary care team, the patient and their family or caregiver. The patient is informed about the management of their health care and takes an active part in the clinical decision-making. *Team-based Care:* Medical care is delivered by an interdisciplinary team, including the patient as a member and the specialist(s) as an expansion of the integrated core team. *Efficient Services:* Through open access, technologies, performance measurements, systems redesign, patient education, and enhanced communications, the patient receives timely, appropriate, and responsive care. All team members work at the top of their competencies to maximize their clinical impact within a time sensitive period. *Comprehensive Car*e: The PACT addresses all medical, behavioral, psychosocial and functional status issues on an ongoing basis. Psychologists and social workers are embedded in the primary care PACT. The PACT involves community partners and providers when necessary, to provide a full spectrum of care. *Continuous Care:* A continuous, longitudinal relationship between the Veteran and PACT provides for all the patient’s health care needs, either directly or collaboratively with specialists. The PACT adjusts over time to the patient’s needs, depending on the complexity of the care and stage of illness. *Communication:* Reliable, accessible, and culturally sensitive communication between the patient and the PACT promotes an honest dialogue without fear of judgment or repercussions and allows the PACT to make informed recommendations, meanwhile, respecting the patient as the locus of control. *Coordination:* The partnership between the patient and PACT allows care to be implemented through a coordinated and active interdisciplinary approach across specialties and settings, facilitated by information technology, access to health information and other means to assure that the patient makes informed choices and gets the agreed upon appropriate care. There is coordination of care between the core PACT and the expanded team that involves specialty consultants, to ensure that high risk transitions are managed appropriately and seamlessly.

### Individual placement and support intervention

IPS is an evidence-based vocational rehabilitation intervention that focuses on the client obtaining and sustaining competitive employment that aligns with their skills, abilities, and preferences, without prevocational training or transitional work assignments [27, 28]. An IPS specialist serves a caseload of up to 20 clients and carries out all phases of employment services, e.g. intake, assessment, job development, job coaching, and follow-along supports in the context of a competitive job. IPS involves the following domains: *Eligibility Based on Client Choice:* IPS embraces the notion of “zero exclusion” whereby clients who want to work are eligible for IPS services; *Personalized Benefits Counseling:* IPS help clients navigate complex systems and obtain personalized information about their VA, Social Security, Medicaid, and other government entitlements; *Rapid Job Search:* IPS Specialists use a rapid job search to help clients obtain jobs directly, rather than starting with pre-employment testing and training; *Systematic Job Development:* IPS Specialists spend most of their time in the community cultivating relationships between their clients and potential employers, working to build an employer network based on clients’ interests; *Competitive Employment:* IPS assists participants to seek and obtain competitive jobs that are consistent with their interests, skills, abilities, and preferences, and that not in sheltered settings; *Integration of IPS and Treatment Team:* IPS is integrated with the treatment team (in this case primary care PACT) and the IPS specialist encourages the client to adhere to treatment in order to achieve employment and recovery goals; *Follow-Along Support:* Individualized follow-along support in the employment and treatment settings is continued for as long as needed.

### Treatment-as-usual vocational rehabilitation services

The Tuscaloosa VA Medical Center provides a broad array of prevocational assessments and vocational rehabilitation services; however, for purposes of this study, treatment consists of the client either obtaining a transitional work assignment within the local VHA setting or working with a vocational rehabilitation specialist who assists with community job placement and brief follow-up support. The rationale for transitional work is the belief that clients with a mental illness need a gradual introduction into regaining work capacity, because of their limited skills and experience, and/or their sensitivity to stress in the competitive work environment. After gaining experience in a protected work setting, it is assumed that clients are more capable of succeeding in competitive employment. The transitional work assignments are set-aside and time-limited and are typically entry-level unskilled positions that are not necessarily matched to the client’s skills and preferences. The vocational rehabilitation specialists provide some guidance for competitive community-based job searches and placement, but they do not provide long-term follow-up after the first competitive job is obtained or the transitional work assignment ends. TAU-VR specialists serve a larger caseload than that of an IPS specialist and are not integrated within a mental health or PACT clinic.

### IPS specialist training and Fidelity monitoring

At the beginning of the study, the IPS Specialist attended a 6-week web-based IPS course sponsored by the IPS Employment Center (affiliated with Westat, Inc.). Throughout the course of the study, the IPS fidelity monitor (RT) holds weekly teleconferences and intermittent on-site visits with the IPS specialists to provide technical assistance. The IPS fidelity monitor conducts a 2-day fidelity monitoring review at least three times per year which includes observation of the IPS specialist in the field during job development and participant interactions, interviews with participants, clinical treatment providers and leadership, and review of the VA electronic medical record. The IPS fidelity monitor evaluates the site using the 25-item Supported Employment Fidelity Scale [29–31]. The IPS fidelity monitor also evaluates the TAU-VR control to ensure that the control group does not receive supported employment (i.e. should score < 55 on the Supported Employment Fidelity Scale). The IPS fidelity monitor provides feedback on the fidelity ratings to the IPS Specialists and investigators.

### Sample size and planned primary outcome analysis

Using SamplePower 3.0 to estimate the statistical power necessary to test the primary hypothesis, the target sample size of 120 participants (60 per group) provides 84% power to detect a 25% or greater absolute difference between groups in the percent of participants achieving ‘steady worker’ primary outcome success status (e.g., 40% in the IPS arm vs. 15% in the control arm), at the .05 level of significance, assuming a 10% attrition. With 60 per group we can lower the power to 0.80 for two sided α = 0.05 with a minimal detectable difference of 40% vs. 17%, respectively.

The primary outcome, defined as the proportion of steady workers, will be analyzed using a logistic regression model to calculate an odds ratio. Weekly employment data values will be summed over the total follow-up period to provide a score for each Veteran. Participants with cumulative scores of ≥26 weeks worked in a competitive job are considered a steady worker primary outcome success and those with cumulative scores < 26 weeks are considered a primary outcome failure. For purposes of primary outcome analysis, missing data will be counted as “not worked.” Adhering to the principle of intent-to-treat, participants may discontinue the treatment intervention, but are encouraged to remain in the study for outcome assessments for the 12-month follow-up period. Analyses of the employment outcomes will also include total time worked (days or weeks), income earned from competitive sources and all sources, and type of jobs held. Total mean time worked will be compared using an analysis of variance (ANOVA) adjusted for site or the Kruskal–Wallis test if the data are not normally distributed.

The effect of treatment on each of secondary and tertiary outcome measures will be analyzed using a longitudinal mixed-effects regression model [32, 33]. The group by time interaction will test for the treatment effect, as we expect the equivalent groups at baseline to diverge over the follow-up period, with the IPS group showing greater gains than the control group. Mixed-effects regression methods assume that data are missing at random and use all available data to estimate the model parameters. In aggregate, there is one statistical model for the primary hypothesis, and there are two for the secondary hypotheses; therefore, there is no compelling reason to adjust the alpha level downward in order to avoid Type I errors. However, to provide assurance and control for multiple comparisons, a sequentially rejective procedure will be conducted to determine statistical significance for the treatment comparisons for secondary outcomes using an overall Type I error of 5% (two-sided) [34].

## Results

**Fig. 1:**
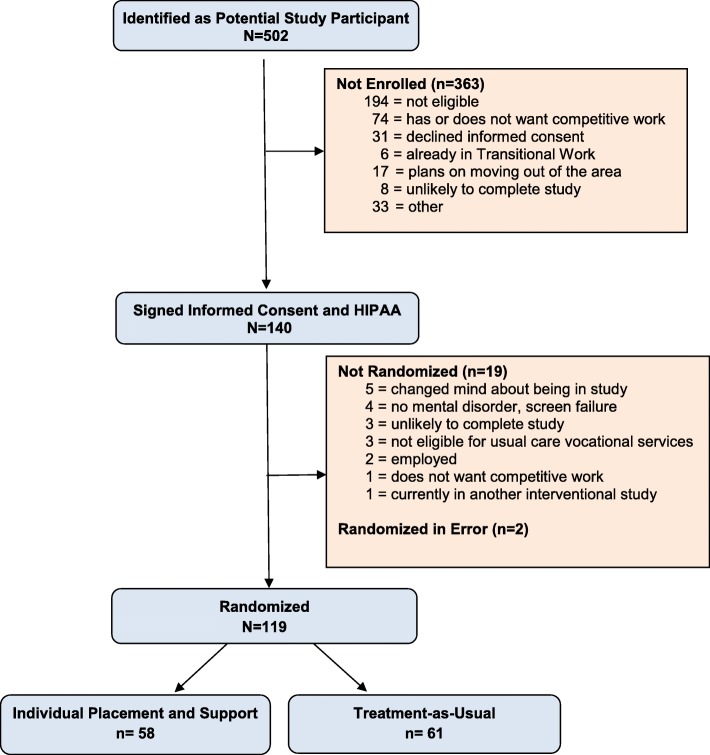
CONSORT

From July 8, 2015 until September 28, 2018, 502 veterans were identified as potential participants, 140 signed informed consent and 119 were randomized to IPS (*n* = 58) or TAU-VR (*n* = 61). The study closed for enrollment on October 16, 2018. Two participants were randomized in error and have been excluded from the randomized sample. Reasons for not being enrolled or randomized are listed in Fig. 1.

As shown in Table 1, the sample is comprised of 17.6% females, 82.4% males, 73.1% African-Americans, 26.1% Whites, and 1.7% participants of Hispanic ethnicity. Participants ranged in age from 23 to 65 years old with an average age of 38.2 (*SD* ± 8.4). Marital status was well distributed between categories. One participant had less than a high school diploma, while 31.1% of participants obtained at least a high school diploma and all others had some college credit (43.7%) or higher (24.3%) education. Most participants had adequate housing (84%), although 17.6% reported inadequate housing. Most participants served in the Army (73.9%), National Guard (23.5%), or Navy (14.3%) with an average time in service of 6.5 (*SD* ± 5.6 years). The periods of service distribution consisted of post-9-11-2001 (80.7%), 1991 through 2001 (10.8%), and Persian Gulf War (19.1%), and 81.7% of participants served in a combat zone.

**Table 1:** Baseline Demographics, Housing Status, and Military History

Variable	IPS (*n =* 58)		TWP (*n =* 61)		Total (*N =* 119)	
	*N*	*%*	*n*	*%*	*n*	*%*
Gender						
Male	49	84.5	49	80.3	98	82.4
Female	9	15.5	12	19.7	21	17.6
Race						
White	15	25.9	16	26.2	31	26.1
African-American	42	72.4	45	73.8	87	73.1
All Other	1	1.7	0	0	1	0.80
Spanish, Hispanic or Latino Ethnicity	1	1.7	1	1.6	2	1.7
Marital Status						
Married	18	31.0	11	18.0	29	24.3
Divorced	19	32.7	19	31.2	38	31.9
Never married	15	25.8	17	27.9	32	26.9
Separated/Cohabitating/Widowed	6	10.3	14	23.0	20	16.8
Education						
Less than high school	1	1.7	0	0	1	0.8
High school diploma	13	22.4	24	39.3	37	31.1
Some college credit	24	41.4	28	45.9	52	43.7
Associate Degree	11	19.0	5	8.2	16	13.4
Bachelor Degree	6	10.3	4	6.6	10	8.4
Master’s or Doctoral Degree	3	5.2	0	0	3	2.5
Branch of Service						
Army	34	58.6	54	88.5	88	73.9
National Guard	11	19.0	17	27.9	28	23.5
Navy	15	25.9	2	3.3	17	14.3
Marine Corp	6	10.3	1	1.6	7	5.9
Air Force	4	6.9	3	4.9	7	5.9
Merchant Marines	1	1.7	0	0	1	0.8
Period of Service						
Persian Gulf War	10	17.2	13	21.3	23	19.1
March 1991–August 2001	14	24.1	14	23.0	28	23.5
Post 9-11-2001	47	81.0	48	78.7	96	80.7
Served in Combat Zone	49	84.5	49	80.3	98	81.7
Housing^a^						
Single Family home	22	37.3	23	44.2	45	37.8
Townhouse/Apartment/Condo	16	27.1	19	36.5	35	29.4
Other Housing	8	13.6	10	16.4	20	16.8
Inadequate Housing^b^						
Transitional housing	7	12.1	6	9.8	13	10.9
Homeless shelter or other	5	8.6	3	4.9	8	6.7
	*M*	*SD*	*M*	*SD*	*M*	*SD*
Length of military service (years)	7.4	6.3	5.6	4.3	6.5	5.6
Length of active duty service (years)	9.6	7.4	9.7	7.3	9.7	7.3
Time at Residence^a^ (months)	20.1	27.8	24.7	57.3	22.4	45.2
Age (years)	37.8	8.4	38.6	8.4	38.2	8.4
Age Range	(23–65)		(24–58)		(23–65)	

*Note.*^a^if reporting adequate housing; ^b^if reporting inadequate housing

As shown in Table 2, most participants were receiving or filing for VA service-connected (SC) disability income (76.5%) with an average total VA service-connected disability rating of 61.0% (*SD* ± 25.9). Only 3 (2.5%) participants were receiving Social Security Disability Income. Participants had been unemployed for an average of 1.4 (*SD* ± 2.3) years and 26.9% held no jobs in the previous 3 years. The longest competitive job held in lifetime averaged 4.7 (*SD* ± 5.3) years.

**Table 2:** Baseline Disability Status, Work History, and Number of Dependents

Variable	IPS (*n =* 58)		TWP (*n =* 61)		Total (*N =* 119)	
	*n*	*%*	*n*	*%*	*n*	*%*
SSDI or SSI						
Yes	1	1.9	2	3.3	3	2.5
No	57	98.3	59	96.7	116	97.5
If no, filing for SSI or SSDI	3	5.2	10	16.4	13	10.9
VA SC Disability Status						
None	13	22.4	13	21.3	26	21.8
Filing for the First Time	7	12.1	10	16.4	17	14.3
Filing for Appeal	1	1.7	1	1.6	2	1.7
Receiving VA SC Disability	25	43.1	27	44.3	52	43.7
Receiving & Filing for Increase	12	20.7	10	16.4	22	18.5
	*M*	*SD*	*M*	*SD*	*M*	*SD*
VA Service-Connected Disability						
Total Percent Disability	68.3	24.1	53.6	25.9	61.0	25.9
Length of current unemployment (years)						
Years	1.1	1.6	1.7	2.7	1.4	2.3
Range (years)	(0–10)		(0–15)		(0–15)	
Duration of longest job in lifetime						
Years	5.4	5.6	4.1	4.9	4.7	5.3
Range (years)	(0–26)		(0–25)		(0–26)	
	*n*	*%*	*n*	*%*	*n*	*%*
Number of jobs held in past 3 years						
Zero	12	20.7	20	32.8	32	26.9
One	13	22.0	14	23.0	27	22.7
Two	16	27.0	11	18.0	27	22.7
Three or more	17	31.0	16	26.0	33	27.7
Financial Dependents						
One	23	40.0	24	39.3	47	39.5
Two	12	20.3	10	16.4	22	18.5
Three or more	23	39.7	27	44.3	50	42.0

*Note.* SSDI = Social Security Disability; SSI = Supplement Security Income

As shown in Table 3, participants had the following diagnoses: past major depression (81.5%), current major depression (70.6%), agoraphobia (15.8%), panic disorder (42.0%), alcohol use disorder within the past year (46.2%), and non-alcohol substance use disorder within the past year (47.0%). Participants had a history of mild (52.2%), moderate (7.6%), or severe (0.8%) traumatic brain injury. On average, participants reported moderate burden of medical comorbidity (CIRS), low self-esteem (RSES) and low quality of life (QOLI).

**Table 3:** Baseline Assessments

Variable	IPS (*n =* 58)		TWP (*n =* 61)		Total (*N =* 119)	
	*n*	*%*	*n*	*%*	*n*	*%*
MINI International Neuropsychiatric Interview						
Major Depression (past)	49	84.5	48	78.7	97	81.5
Major Depression (current)	43	74.1	41	67.2	84	70.6
Major Depression (recurrent)	25	43.1	22	36.1	47	39.5
Agoraphobia (current)	7	12.1	13	21.3	19	15.8
Panic (lifetime)	24	41.4	26	42.6	50	42.0
Social Anxiety (current)	6	10.3	8	13.1	14	11.8
Obsessive Compulsive (current)	0	0	2	3.3	2	1.7
Alcohol Abuse/Dependence (past year)	29	44.8	26	36.1	55	46.2
Substance Abuse (past year)	15	25.9	13	21.3	28	23.5
Substance Dependence (past year)	14	24.1	14	23.0	28	23.5
Ohio State University Traumatic Brain Injury Worst Injury Level for Participant						
Mild TBI	18	23.7	12	18.0	30	52.2
Moderate TBI	3	5.1	6	9.8	9	7.6
Severe TBI	0	0	1	1.6	1	0.8
	*M*	*SD*	*M*	*SD*	*M*	*SD*
Cumulative Illness Rating Scale						
Total Score	4.4	2.6	4.2	0.5	4.4	2.6
Severity Index	1.7	0.5	1.7	0.5	1.7	0.5
Quality of Life Inventory Score	0.3	1.7	0.9	2.1	0.6	1.9
Rosenberg Self Esteem Scale	17.8	5.9	19.1	5.4	18.5	5.7

*Note.* Quality of Life Inventory score range − 6 to 6 and ≥ 0 = higher life satisfaction; Cumulative Illness Rating Scale score range 0–56; Rosenberg Self-Esteem Scale score range 10–40 and < 25 = low self-esteem. TBI = Traumatic Brain Injury

Overall, the distribution of baseline demographics and clinical characteristics was balanced and nonsignificant between groups, with exception on marital status. A higher percentage of IPS participants were married (31%) while a higher percentage of TAU-VR group were separated or cohabitating (23%).

### Study timeline and modifications to the protocol

Although the study was officially launched in April 2015 and recruitment activities started in mid-June 2015, the IPS specialist position was not officially filled until mid-July 2015 and the first participant was randomized the following week. Recruitment rates were lower than expected during the first year (61% of goal) and the following modifications were made to the protocol in year 1. We initially restricted the study to one PACT, called the Transition Center, that exclusively served veterans who had returned from Iraq or Afghanistan deployments within the past five years. Despite assertive outreach and strong clinical provider engagement, this clinic did not yield an adequate number of unemployed veterans who were interested in competitive employment and vocational rehabilitation services. Thus, we expanded IPS services to other primary care PACT clinics and changed the eligibility criteria to include veterans from all U.S. military conflicts spanning from 1990 Desert Storm to the present. In addition, we experienced a downsizing of our TAU-VR transitional work programs and needed to make clarifications to the protocol that the randomization to the existing TAU-VR services could include modalities such as prevocational counseling and community employment services. These changes restored the recruitment flow; however, the enrollment period had to be extended to allow time for meeting an adequate sample size.

## Discussion

Given the substantial challenges many veterans with mental health issues face when transitioning successfully into community life and sustaining gainful employment, effective vocational rehabilitation employment services are critical to preventing long-term disability and rising societal costs [35–37]. VA usual-care vocational services are not fully meeting the needs of veterans with non-serious mental illnesses and thus, more effective and engaging strategies are needed [12–14]. In addition, the frequency and compensation amounts for disability benefits in veterans have increased significantly over the past two decades [38], and the percentage of disability rating (i.e. degree of functional impairment 0 to 100%) has been shown to be an important determinant of premature mortality [39]. These facts, as reflected by the degree of disability rating in our sample, make it ever more important and urgent to test innovations and provide evidence-based vocational services. This study evaluates a novel approach to the delivery of employment services, namely integrating evidenced-based IPS within the primary care PACT. In addition, the study widens the aperture of services to veterans with general mental health issues, such as major depressive, anxiety, PTSD, and substance use disorders.

Regarding sustaining steady competitive work, we do not know whether patients with general mental health diagnoses benefit more from having IPS offered in a primary care PACT compared to being referred to TAU-VR services that include transitional work assignments and community-based employment services. In addition, we do not know for certain whether high fidelity IPS can be achieved in a primary care PACT setting. For these reasons, a randomized-controlled trial was justified. The results will inform stakeholders as to whether more intensive employment services delivered in a primary care setting are effective and feasible. If we find no difference between groups, we would need to take a step back to consider whether it is possible to deliver IPS in a PACT setting or whether patients diagnosed with nonpsychotic disorders are just as likely to thrive when provided less intensive usual-care services.

### Why would we want to offer IPS in a primary care setting?

Unemployment is linked to declining physical health, excess mortality and increased health care utilization and costs [40, 41]. A primary care clinic may be an appropriate place to engage patients who are unemployed and considering return to competitive employment. IPS embraces the notion of zero exclusion, a principle whereby veterans who want to work are deemed eligible and are not denied IPS services due to psychiatric diagnosis, cognitive impairment, substance use, legal history, etc. Usual care vocational rehabilitation practices often exclude persons still experiencing active symptoms or physical limitations, and suggest they stay engaged in their prescribed treatment until they are “ready” to work. The integration of IPS within an effective, respectful treatment team is essential. Most traditional vocational rehabilitation models are compartmentalized and kept separate from the clinical treatment providers. A well-implemented IPS design has integrated relationships between the IPS specialist and the primary PACT providers which can assist veterans to access timely and relevant medical or psychological supports to enhance their recovery and return to work.

In addition, earlier access to services in a primary care setting may be an advantage given that veterans often resist a referral to a mental health clinic due to the stigma of having a mental illness and fear that a diagnosis will hinder their ability to get a job. Employment is often the “hook” through which many clients get more invested in their treatment and recovery. Competitive employment aligns with veterans’ priorities of having the resources needed to take care of themselves and their families, feel good about themselves, integrate in the community, and regain dignity and self-respect. A secondary benefit of a systematic IPS job development process is that the veteran achieves employment while also accessing clinical supports to address their physical and mental health needs, including treatment to manage depression, anxiety, PTSD, insomnia, and addictions.

### How can IPS be delivered in a PACT setting?

When IPS is well integrated within the PACT, the IPS employment specialist partners closely with the clinical providers to assist the veteran in overcoming the mental health obstacles so that they may be more fully supported and included in the workforce. Additionally, this model allows the Veteran to simultaneously adhere to effective medical and mental health interventions. The IPS specialist needs to find creative ways to communicate and collaborate with busy primary care providers who have extremely large caseloads and a limited amount of time per appointment to address the patient’s needs while in the clinic.

A prerequisite for successful implementation of IPS within PACT is a cohesive, integrated team approach that provides many opportunities for communication and collaboration around the intersection of the treatment objectives and the employment plan. Weekly team meetings or “huddles” are commonly the proposed format for this approach. These huddles or meetings between the clinical staff and the IPS Employment Specialist provide opportunity for defining common, clinically sound messages and approaches which provide the tools for the veterans to overcome their health challenges and build resources to become self-sufficient, independent members of their communities.

## Conclusions

IPS is rapidly expanding to patient populations other than those with serious mental illness [42]. For example, studies have included people with substance use disorder, musculoskeletal or neurological disorders, spinal cord injuries, and PTSD. Although modifications in the IPS approach are often needed to fit the particular needs of the person with a disability other than serious mental illness, the core principles of IPS remain consistently important in attaining high employment outcomes and improvement in quality of life.

An intervention, such as IPS, that directly addresses the occupational recovery of veterans and can be delivered within the frontline service of a PACT, has the potential to improve veterans’ employment outcomes, personal income, and quality of life, while also increasing income tax revenue and offsetting or reversing disability costs. The renewed status and sense of identity that comes with being employed and making meaningful contributions to the home, family and society, can reduce the stigma of disability, lower symptoms of depression and anxiety, and reduce the number and severity of crisis events. The outcomes of this study may lead to a shift in rehabilitation services within the VA and improve the lives of thousands of veterans who would otherwise have difficulty with reintegration and be pushed to the unhealthy and dangerous margins of society.

## Data Availability

The datasets generated during the current study are not publicly available to be shared nor can the dataset be retrospectively approved for data sharing or for inclusion in a data repository due to the fact that at the time of initial approval and study initiation, data sharing plan was not proposed nor included in the informed consent. The aggregated results will be made available in clinicaltrials.gov under the study identifier NCT02400736. Aggregated datasets are available from the corresponding author on reasonable request to the corresponding author, if approved by the local institutional review board accompanied by an approved data use agreement.

## Abbreviations

ANOVA: Analysis of variance
CRC: Clinical Research Coordinator
CRIS: Community Reintegration of Service Members
CSQ-8: Client Satisfaction Questionnaire-8
IPS: Individual Placement and Support
MINI: MINI International Neuropsychiatric Interview
PACT: Patient Aligned Care Team
PTSD: Posttraumatic stress disorder
QOLI: Quality of Life Inventory
RSES: Rosenberg Self-Esteem Scale
SCL-90-R: Symptom Checklist-90-Revised
SD: Standard deviation
SMI: Serious mental illness
SSI/SDI: Supplemental Security Income/Social Security Disability Income
SSTS: Sheehan Suicidality Tracking Scale
TAU-VR: treatment-as-usual vocational rehabilitation
VA: U.S. Department of Veterans Affairs
VHA: Veterans Health Administration
